# Extracellular vesicles derived from mesenchymal stem cells alleviate neuroinflammation and mechanical allodynia in interstitial cystitis rats by inhibiting NLRP3 inflammasome activation

**DOI:** 10.1186/s12974-022-02445-7

**Published:** 2022-04-06

**Authors:** Chi Zhang, Yong Huang, Fubing Ouyang, Minzhi Su, Wenbiao Li, Jialiang Chen, Hengjun Xiao, Xiangfu Zhou, Bolong Liu

**Affiliations:** 1 Department of Urology, The Third Affiliated Hospital, Sun Yat-Sen University, 600 Tianhe Road, Guangzhou, 510630 China; 2 Department of Neurology, The First Affiliated Hospital, Sun Yat-Sen University, 74 Zhongshan Road 2, Guangzhou, 510080 China; 3 Department of Rehabilitation, The Third Affiliated Hospital and Lingnan Hospital of the Sun Yat-Sen University, 2693 Kaichuang Road, Guangzhou, 510700 China

**Keywords:** Interstitial cystitis/bladder pain syndrome, Mesenchymal stem cell, Extracellular vesicle, NLRP3 inflammasome, Neuroinflammation

## Abstract

**Background:**

Neuroinflammation in spinal dorsal horn (SDH) plays an important role in the pathogenesis of interstitial cystitis/bladder pain syndrome (IC/BPS). Mesenchymal stem cell-derived extracellular vesicles (MSC-EVs) exert potent anti-inflammatory activities in the treatment of various diseases. This study aimed to determine the therapeutic effects of MSC-EVs on IC and furtherly investigate the potential mechanism to attenuate neuroinflammation.

**Methods:**

Female IC rat model was established by intraperitoneal injection of cyclophosphamide (50 mg/kg, every 3 days for 3 doses). Inhibition of NLRP3 inflammasome was performed by intraperitoneal injection of MCC950 (10 mg/kg). MSC-EVs were isolated from the culture supernatants of human umbilical cord derived MSCs using ultracentrifugation, and then injected intrathecally into IC rats (20 μg in 10 μl PBS, every other day for 3 doses). Suprapubic mechanical allodynia was assessed using up-down method with von Frey filaments, and micturition frequency was examined by urodynamics. The expression of NLRP3 inflammasome components (NLRP3 and Caspase-1), glial cell markers (IBA-1 and GFAP), proinflammatory cytokines (TNF-α, IL-1β, IL-6 and IL-18) and TLR4/NF-κB signal pathway (TLR4, p65 NK-κB and phospho-p65 NK-κB) in L6–S1 SDH was measured by Western blot analysis. The cellular localization of NLRP3 in SDH was detected using immunofluorescence co-staining.

**Results:**

NLRP3 inflammasome was activated in neurons in SDH of IC rats. NLRP3 inflammasome activation contributed to activation of glial cells and process of spinal neuroinflammation in IC rats, and was related to suprapubic mechanical allodynia and frequent micturition. Intrathecal injection of MSC-EVs alleviated suprapubic mechanical allodynia and frequent micturition in IC rats, restrained activation of glial cells and attenuated neuroinflammation in SDH. In addition, MSC-EV treatment significantly inhibited activation of both NLRP3 inflammasomes and TLR4/NF-κB signal pathway.

**Conclusions:**

NLRP3 inflammasome activation is involved in the neuroinflammation of IC. Intrathecal injection of MSC-EVs alleviates neuroinflammation and mechanical allodynia in IC by inhibiting the activation of NLRP3 inflammasome, and TLR4/NF-κB signal pathway may be the potential regulatory target.

## Introduction

Interstitial cystitis/bladder pain syndrome (IC/BPS) is a chronic condition predominant in females, characterized by pelvic pain related to bladder filling, accompanied by lower urinary tract symptoms such as urinary frequency and urgency, but in the absence of any identifiable cause [1, 2]. The prevalence of IC is estimated to be 300 per 100,0000 women [3], and its intractable chronic pain symptom is a severe detriment to patients’ quality of life [4]. The precise etiology of IC is still ambiguous, and multiple theories have been suggested, including endothelial permeability, subclinical infection, autoimmunity, neurologic abnormality and genetic susceptibility [5, 6]. Owing to the complex pathogenesis, current therapies for IC are not satisfactory and only provide partial relief [2, 7], highlighting the urgent need to develop new effective treatment options for IC. Our previous studies have demonstrated that neuroinflammation in the spinal dorsal horn (SDH) contributes to bladder hypersensitivity and plays a pivotal role in the pathogenesis of IC [8–10]. Therefore, targeting the neuroinflammation in SDH may be a novel strategy for IC treatment.

Inflammasomes are a group of intracellular multimeric protein complexes assembled by pattern recognition receptors that can detect pathogen- or damage-associated molecular patterns and activate inflammatory reaction [11]. The nucleotide binding and oligomerization domain-like receptor family pyrin domain-containing 3 (NLRP3) inflammasome is the most well-characterized member, which consists of sensor protein NLRP3, adaptor protein apoptosis-associated speck-like protein containing a caspase recruitment domain, and effector enzyme pro-caspase-1 [12]. Upon sensing danger stimuli, the NLRP3 inflammasome is assembled and activated to trigger the release of proinflammatory cytokines IL-1β and IL-18, subsequently participating in the initiation and amplification of the inflammatory reaction [13]. The aberrant activation of NLRP3 inflammasome is demonstrated to be involved in traumatic brain injury [14], neuropathic pain [15], Alzheimer’s disease [16], etc., indicating that NLRP3 inflammasome is crucial for the process of neuroinflammation. Thus, it is necessary to elucidate the relationship between NLRP3 inflammasome and spinal neuroinflammation in IC.

Mesenchymal stem cells (MSCs) possess potent anti-inflammatory and immunomodulatory activities [17, 18], and stem cell therapy based on MSCs is considered a promising alternative for diseases associated with inflammation [19, 20]. Nowadays it is generally accepted that the therapeutic efficacy of MSCs is mainly attributed to their paracrine factors rather than cell differentiation and replacement of damaged cells [21, 22]. Furthermore, increasing evidence has revealed that MSCs perform most of the paracrine effects by releasing extracellular vesicles (EVs) [23], membrane-enclosed vesicles with a diameter of 50–1000 nm that are secreted by almost all types of cells [24, 25]. EVs carry specific cellular components of the source cells, and the proteins, nucleic acids and metabolites delivered by EVs into recipient cells can alter their biological response [26]. As a cell-free therapeutic tool, MSC-derived EVs (MSC-EVs) not only own high stability, but also avoid risk of immune rejection, tumor formation and vascular obstruction, showing overwhelming superiority to MSCs [27]. MSC-EVs, especially the small EVs (50–200 nm in diameter) [28], have been reported to exert remarkable anti-inflammatory activities and achieved favorable effects in multiple preclinical models [29–31]. However, whether MSC-EVs are effective for IC and what is the underlying mechanism have not yet been investigated.

Based on the above evidence, in this study, we have focused on the neuroinflammation of IC, and explored whether MSC-EVs alleviated neuroinflammation and mechanical allodynia in a cyclophosphamide-induced cystitis rat model by regulation of NLRP3 inflammasome activation, aiming to provide a new avenue for IC treatment.

## Methods

### Animal modeling and grouping

Ninety-six female Sprague-Dawley rats (200–220 g) were used in this study. All the rats were purchased from the Laboratory Animal Center of Sun Yat-sen University, and the animal procedures were approved by the Institutional Animal Care and Use Committee of Sun Yat-sen University. Cyclophosphamide (Sigma-Aldrich, USA) was used to establish IC rat model as previously described [32]. Briefly, cyclophosphamide (50 mg/kg) was intraperitoneally injected every 3 days for 3 doses.

In the first part of the experiment, we applied a selective NLRP3 inflammasome inhibitor, MCC950 [33], to explore the role of NLRP3 inflammasome in the spinal neuroinflammation of IC, and three groups were included: (1) Control, (2) IC, (3) MCC950 (*n* = 16 per group). Control group was normal rats. IC group and MCC950 group were intraperitoneally injected with cyclophosphamide. On the second day after the first dose of cyclophosphamide, MCC950 group began to receive intraperitoneal injection with MCC950 (10 mg/kg; MedChemExpress, USA), which was injected every 3 days until the end of the experiment. IC group was intraperitoneally injected with the same amount of PBS.

In the second part of the experiment, three groups were included to verify the therapeutic effects of MSC-EVs on IC: (1) Control, (2) IC, (3) MSC-EV (n = 16 per group). Control group was normal rats. IC group and MSC-EV group were intraperitoneally injected with cyclophosphamide as mentioned above. The day after the last dose of cyclophosphamide, MSC-EV group received intrathecal injection of MSC-EVs (20 μg in 10 μl PBS) every other day for 3 doses, and IC group was intrathecally injected with the same amount of PBS.

### MSC-EVs isolation and identification

MSC-EVs were isolated from the culture supernatants of human umbilical cord derived MSCs. The supernatants were obtained from Biotherapy Center of the Third Affiliated Hospital, Sun Yat-Sen University, and contained no serum components. EVs isolation was carried out using ultracentrifugation as previously described [34]. Briefly, MSC culture supernatants were subjected to successive centrifugations at 300×*g* (10 min, 4 °C), 2000×*g* (10 min, 4 °C), and 10,000×*g* (30 min, 4 °C). The pellets were discarded, and the supernatants were subjected to ultracentrifuged at 100,000×*g* for 70 min at 4 °C (SW28Ti rotor, Beckman Coulter, USA). The pellets were washed in phosphate buffered saline (PBS) and followed by a second ultracentrifugation at 100,000×*g* for 70 min at 4 °C. The pellets (EVs) were then resuspended in PBS. We observed the morphology of MSC-EVs by transmission electron microscopy (TEM) (JEM-1200EX, JEOL, Japan), and detected the particle size distribution by dynamic light scattering (DLS) (Litesizer 500, Anton Paar, Austria). EV protein markers (CD9, CD63, CD81 and ALIX) were measured by Western blot analysis.

### Intrathecal injection

Intrathecal injection was performed as our previous study described [8, 32]. Under isoflurane inhalation anesthesia, a 25-gauge needle connected to Hamilton syringe was punctured into spinal canal in the intervertebral space between L5 and L6. A tail-flick reaction indicated a successful puncture, and then MSC-EVs or PBS was injected. After injection, the needle remained at the puncture site for over 15 s to ensure reagent delivery and avoid leakage.

### Mechanical allodynia assessment

Up-down method with von Frey filaments (Aesthesio, USA) was used to test the withdrawal threshold in the suprapubic region of rats, which has been proven to be an effective method for assessing mechanical allodynia in cystitis rat models [35]. Before the test, rats were acclimatized to the test cage environment for 30 min. Then a series of von Frey filaments with different strengths (1.4 g, 2 g, 4 g, 6 g, 8 g and 15 g) were used to stimulate the suprapubic region in turn. Each stimulus lasted 6–8 s, and each strength was repeated 3 times with an interval of 5 min. A positive response was classified as licking or scratching the stimulated site, or flinching or arising from the stimulation. Mechanical allodynia was assessed every 3 days throughout the experiment.

### Urodynamic evaluation

Urodynamic evaluation was performed according to our previously described methods [36]. Briefly, after restraining the rat, the bladder was emptied using manual abdominal pressure. A PE-50 catheter was advanced retrograde into the bladder and connected to a pressure transducer (BL-420F, Taimeng Technology, China) in line with an infusion pump. Sterile normal saline was infused at 6 mL/h and intravesical pressure was continuously recorded with BL New Century 2.1 software (Taimeng Technology, China). After voiding cycles stabilized (typically three to four cycles), an additional 30 min was recorded for quantitative analysis.

### Western blot analysis

The L6–S1 SDH was harvested and total protein was extracted using RIPA lysis buffer (CW2333, CWBIO, China) containing proteinase and phosphatase inhibitors. Protein concentration was determined using Pierce BCA Protein Assay Kit (23227, Thermo Fisher Scientific, USA). Protein samples were separated by sodium dodecyl sulfate polyacrylamide gel electrophoresis and transferred onto polyvinylidene fluoride membranes. After blocking with 5% bovine serum albumin, the membranes were incubated with following primary antibodies: CD9 (ab92726, 1:1000; abcam, USA), CD63 (ab68418, 1:1000; abcam, USA), CD81 (ab109201, 1:1000; abcam, USA), ALIX (ab117600, 1:1000; abcam, USA), TNF-α (BS1857,1:1000; Bioworld Technology, USA), IL-1β (ab9722, 1:1000; abcam, USA), IL-6 (DF6087, 1:1000; Affinity Biosciences, USA), IBA-1 (ab5076, 1:1000; abcam, USA), GFAP (3670, 1:1000; Cell Signaling Technology, USA), NLRP3 (NBP2-12446, 1:1000; Novus Biologicals, USA), Caspase-1 (sc-56036, 1:500; Santa Cruz, USA), IL-18 (ab191860, 1:1000; abcam, USA), TLR4 (AF7017, 1:1000; Affinity Biosciences, USA), p65 NK-κB (ab16502, 1:1000; abcam, USA), phospho-p65 NK-κB (Ser311) (AF3389, 1:1000; Affinity Biosciences, USA) and GAPDH (T0004, 1:5000; Affinity Biosciences, USA). And then membranes were incubated with secondary antibodies conjugated with horseradish peroxidase. Protein bands were detected using an enhanced chemiluminescence kit (WBKLS0500, Millipore, USA) and band density was quantified by Image J software (National Institutes of Health, Bethesda, USA).

### Immunofluorescent staining analysis

Under pentobarbital sodium (50 mg/kg) anesthesia, the rats were perfused with normal saline and then L6–S1 spinal cord was harvested. Tissue specimens were fixed with 4% paraformaldehyde and then dehydrated with 30% sucrose. After embedding with optimal cutting temperature compound, tissue specimens were sectioned at 20 μm thickness. Sections were blocked with 5% bovine serum albumin and incubated with primary antibodies including NLRP3 (NBP2-12446, 1:100; Novus Biologicals, USA), NeuN (MAB377, 1:200; Millipore, USA), GFAP (3670, 1:300; Cell Signaling Technology, USA) and OX-42 (MCA275G, 1:100; Bio-Rad, USA). Then sections were incubated with secondary antibodies conjugated with Cy3 or FITC, and mounted with antifade mountants (containing DAPI). Images were captured using an inverted fluorescence microscope (EVOS FL, Thermo Fisher Scientific, USA) and fluorescent colocation was quantified by Image J software (National Institutes of Health, Bethesda, USA).

### Statistical analysis

Statistical analyses were performed using IBM SPSS Statistics 23.0 (IBM Corporation, USA). All data were expressed as mean ± standard deviation. One-way analysis of variance followed by a Student–Newman–Keuls post hoc test for multiple comparisons was used as appropriate. Two-tailed *P*-value < 0.05 was considered statistically significant.

## Results

### NLRP3 inflammasome is activated in neurons in the SDH of IC rats

**Fig. 1:**
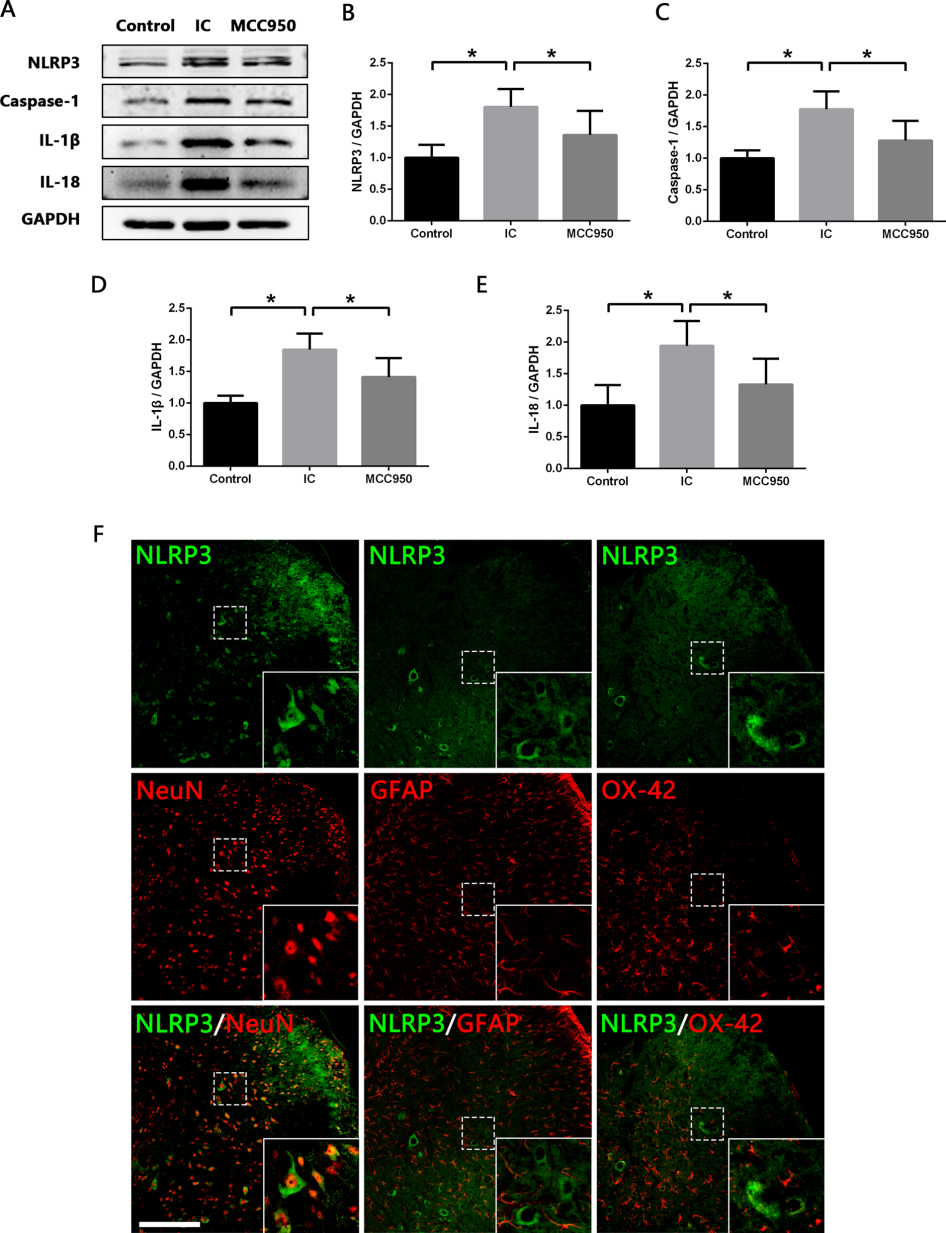
NLRP3 inflammasome is activated in neurons in SDH of IC rats, and MCC950 inhibits the NLRP3 inflammasome activation. **A**–**E** Western blot analysis showing that expression levels of NLRP3, Caspase-1, IL-1β and IL-18 were significantly increased in SDH of IC rats compared with normal rats, and MCC950 treatment significantly decreased expression levels of NLRP3, Caspase-1, IL-1β and IL-18 in SDH of IC rats. *n* = 8 per group. *P < 0.05. **F** Immunofluorescence co-staining showing that NLRP3 was colocalized predominantly with NeuN (neuron marker), but scarcely with GFAP (astrocyte marker) or OX-42 (microglia marker) in the SDH. Scale bars = 200 μm

We first analyzed the expression levels of NLRP3 inflammasome components (NLRP3 and Caspase-1) in the SDH of normal and IC rats. Western blot analysis revealed that NLRP3 and Caspase-1 expression were significantly increased in IC group compared with Control group (Fig. 1A–C). In addition, similar trends were observed in the levels of IL-1β and IL-18 (Fig. 1A, D, E), inflammatory cytokines mediated by NLRP3 inflammasome. Immunofluorescence co-staining and colocation analysis were then performed to determine the cell source of NLRP3 inflammasome in the SDH. The results confirmed that NLRP3 was mainly expressed in NeuN-positive neurons, and approximately 42% of neurons in the SDH expressed NLRP3 (Fig. 1F). While NLRP3 was scarcely colocalized with GFAP-positive astrocytes or OX-42-positive microglia (Fig. 1F). It was indicated that neuron-derived NLRP3 inflammasome activation might be involved in the spinal neuroinflammatory of IC.

### NLRP3 inflammasome contributes to the activation of glial cells and process of neuroinflammation in the SDH of IC rats

To explore the role of NLRP3 inflammasome in the spinal neuroinflammation of IC, MCC950, a selective NLRP3 inflammasome inhibitor, was administrated to IC rats. As shown in Fig. 1A–E, Western blot analysis revealed that expression levels of NLRP3, Caspase-1, IL-1β and IL-18 were significantly reduced in MCC950 group compared to IC group, indicating the potent inhibition of MCC950 on NLRP3 inflammasome activation.

**Fig. 2:**
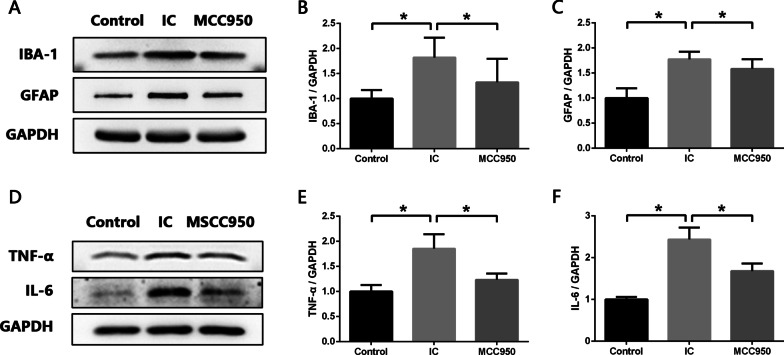
NLRP3 inflammasome contributes to activation of glial cells and process of neuroinflammation in SDH of IC rats. **A**–**C** Western blot analysis showing that expression levels of IBA-1 (microglia marker) and GFAP (astrocyte marker) were significantly increased in SDH of IC rats compared with normal rats, and MCC950 treatment significantly decreased expression levels of IBA-1 and GFAP in SDH of IC rats. **D**–**F** Western blot analysis showing that expression levels of proinflammatory cytokine TNF-α and IL-6 were significantly increased in SDH of IC rats compared with normal rats, and MCC950 treatment significantly decreased expression levels of TNF-α and IL-6 in SDH of IC rats. *n* = 8 per group. *P < 0.05

Microglia and astrocytes are two important glial cells that participate actively in the process of neuroinflammation by releasing multiple proinflammatory cytokines, such as TNF-α, IL-1β and IL-6 [37, 38]. Western blot analysis revealed remarkably increased expression of IBA-1 (microglia marker) and GFAP (astrocyte marker) in the SDH of IC rats compared with normal rats (Fig. 2A–C). However, after MCC950 administration, the expression levels of IBA-1 and GFAP were significantly reduced (Fig. 2A–C), indicating the activation of glial cells was restrained by MCC950. Furthermore, we detected whether MCC950 reduced the levels of proinflammatory cytokines in the SDH of IC rats. Western blot results showed that besides IL-1β, the levels of TNF-α and IL-6 were also upregulated in the SDH of IC rats, and MCC950 administration significantly diminished the TNF-α and IL-6 upregulation (Fig. 2D–F). Therefore, it was confirmed that NLRP3 inflammasome activation contributed to the process of spinal neuroinflammation in IC.

### NLRP3 inflammasome activation is related to the mechanical allodynia and frequent micturition in IC rats

**Fig. 3:**
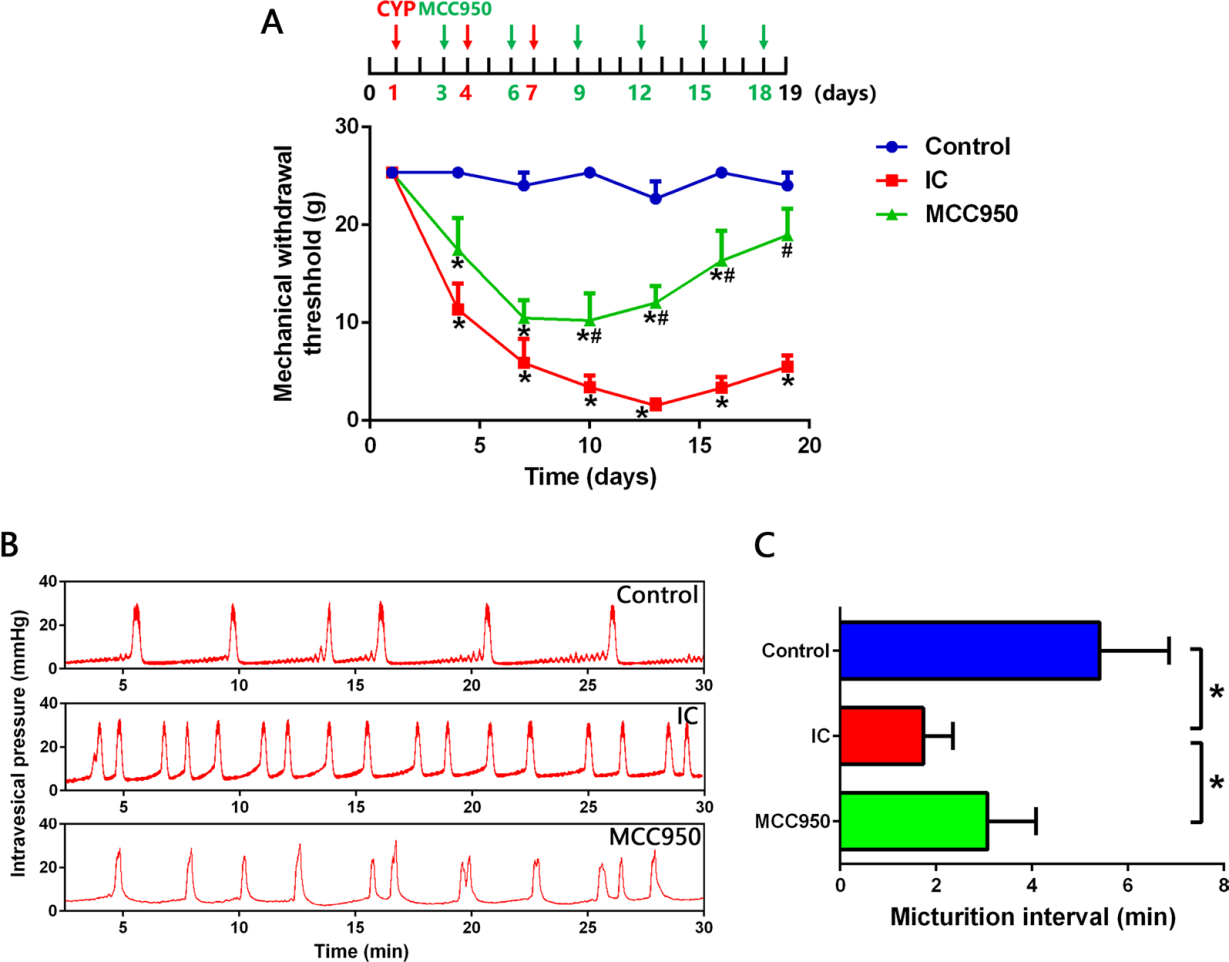
NLRP3 inflammasome activation is related to mechanical allodynia and frequent micturition in IC rats. **A** von Frey test showing that suprapubic mechanical withdrawal threshold of IC rats was significantly lower than normal rats, and MCC950 treatment significantly raised suprapubic mechanical withdrawal threshold of IC rats. *n* = 10 per group. **B**, **C** Urodynamic evaluation showing that IC rats exhibited significantly shorter micturition interval than normal rats, and MCC950 treatment significantly prolonged the micturition interval of IC rats. *n* = 6 per group. *P < 0.05

As shown in Fig. 3A, during the first two administrations of MCC950, mechanical withdrawal threshold of MCC950 group was slightly enhanced compared with IC group, but the differences were not statistically significant. Nevertheless, after the third dose of MCC950, the mechanical withdrawal threshold was significantly enhanced and the effect was maintained until the end of the experiment. Frequent micturition of IC is a symptom related to the pain caused by bladder filling [1]. Urodynamic results showed that rats in IC group exhibited significantly shorter micturition interval than normal rats. After MCC950 administration, the micturition interval was significantly prolonged and the frequent urination was alleviated (Fig. 3B, C). These results indicated that NLRP3 inflammasome activation is closely related to the mechanical allodynia and frequent micturition in IC, and inhibition of NLRP3 inflammasome was an effective approach for IC treatment.

### Characterization of MSC-EVs

**Fig. 4:**
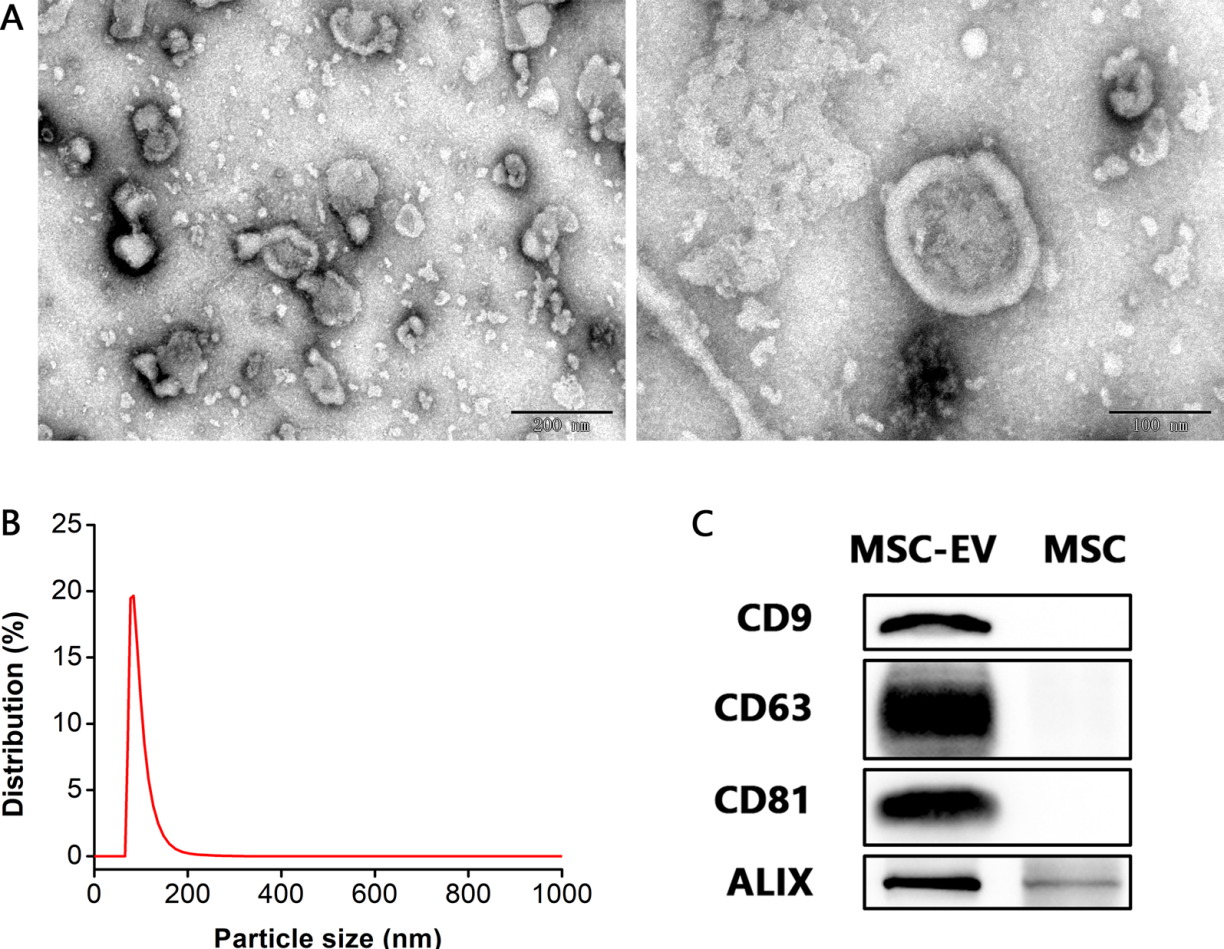
Characterization of MSC-EVs. **A** TEM showing that MSC-EVs had a saucer-like structure. Scale bars = 200 nm (left) and 100 nm (right). **B** Particle size distribution by DLS showing that the majority of MSC-EVs were 80–140 nm in diameter, and the most abundant particle size was 83.97 ± 11.05 nm (*n* = 6). **C** Western blot analysis showing that EV protein markers (CD9, CD63, CD81 and ALIX) were enriched in MSC-EVs compared with MSCs

To investigate the therapeutic effects of MSC-EVs on IC, we first isolated MSC-EVs from the culture supernatants of MSCs, and TEM, nanoparticles analysis by DLS and Western blot analysis were performed to identify the obtained EVs. TEM showed that MSC-EVs had a saucer-like structure (Fig. 4A). DLS showed that the majority of MSC-EVs were 80–140 nm in diameter, and the most abundant particle size was 83.97 ± 11.05 nm (Fig. 4B). Western blot analysis revealed that EV-associated protein markers were enriched in MSC-EVs compared with MSCs, such as transmembrane proteins CD9, CD63 and CD81, and cytosolic protein ALIX (Fig. 4C). These data indicated that the vesicles we obtained from MSC culture supernatants were EVs, and mainly small EVs (50–200 nm in diameter).

### MSC-EVs alleviate the mechanical allodynia and frequent micturition in IC rats

**Fig. 5:**
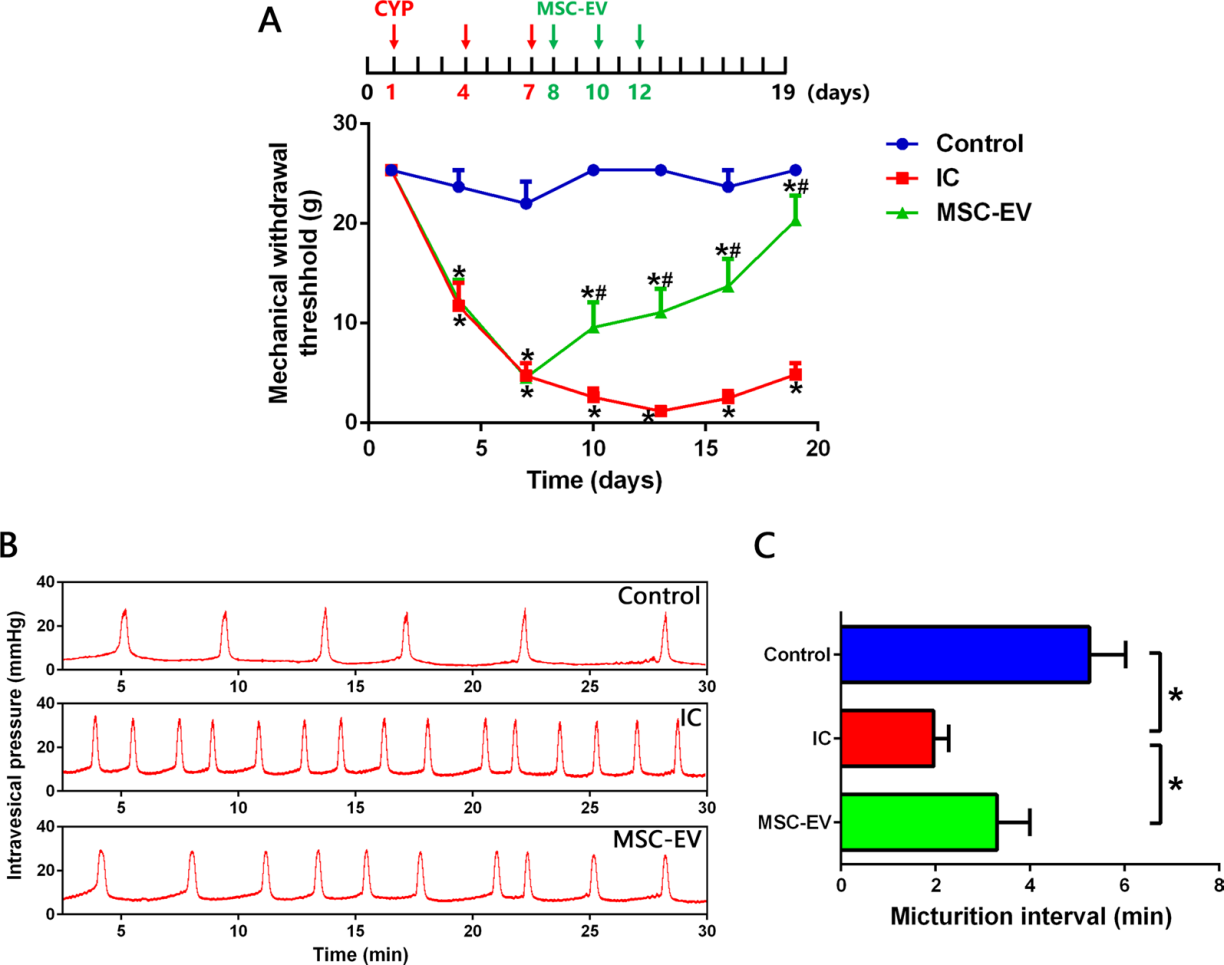
MSC-EVs alleviate mechanical allodynia and frequent micturition in IC rats. **A** von Frey test showing that intrathecal injections of MSC-EVs significantly raised suprapubic mechanical withdrawal threshold of IC rats. **B**, **C** Urodynamic evaluation showing that MSC-EV treatment significantly prolonged the micturition interval of IC rats. *n* = 8 per group. *P < 0.05

MSC-EVs were then injected intrathecally into IC rats to verify their therapeutic effects. As shown in Fig. 5A, the first injection of MSC-EVs reversed the decrease in mechanical withdrawal threshold of IC rats, and the additional two doses of MSC-EVs maintained and strengthened this effect, significantly alleviating the mechanical allodynia of IC rats. Additionally, MSC-EV treatment also significantly extended the micturition interval, alleviating the frequent micturition of IC rats (Fig. 5B, C).

### MSC-EVs restrain the activation of glial cells and attenuate neuroinflammation in the SDH of IC rats

**Fig. 6:**
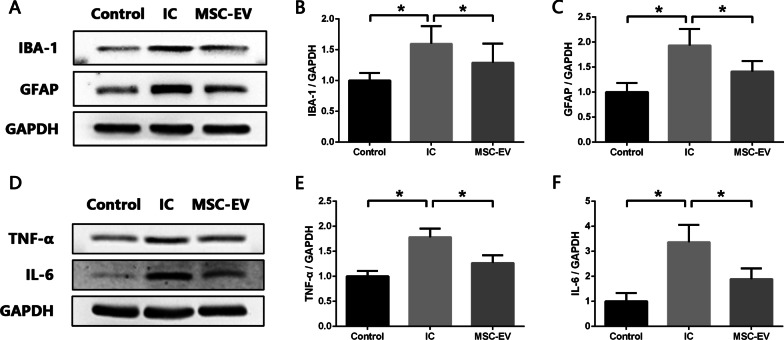
MSC-EVs restrain activation of glial cells and attenuate neuroinflammation in SDH of IC rats. **A**–**C** Western blot analysis showing that intrathecal injection of MSC-EVs significantly decreased expression levels of IBA-1 (microglia marker) and GFAP (astrocyte marker) in SDH of IC rats. **D**–**F** Western blot analysis showing that MSC-EV treatment significantly decreased expression levels of proinflammatory cytokine TNF-α and IL-6 in SDH of IC rats. *n* = 8 per group. *P < 0.05

The effect of MSC-EVs on spinal neuroinflammation in IC rats was then explored using Western blot analysis. As expected, the results showed that MSC-EV treatment significantly decreased the expression of IBA-1 and GFAP in the SDH of IC rats (Fig. 6A–C). Consistent with the inhibition of glial cell activation, levels of TNF-α and IL-6 were also reduced significantly (Fig. 6D–F), suggesting that MSC-EVs could attenuate the neuroinflammation in the SDH of IC rats.

### MSC-EVs inhibit the activity of NLRP3 inflammasome and TLR4/NF-κB signal pathway in the SDH of IC rats

**Fig. 7:**
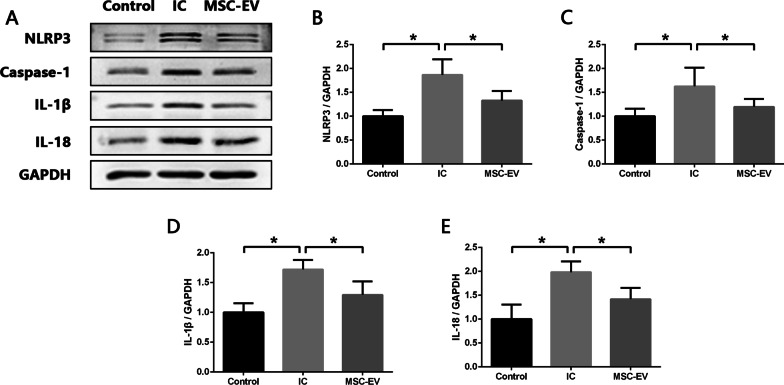
MSC-EVs inhibit activity of NLRP3 inflammasome in SDH of IC rats. **A**–**E** Western blot analysis showing that intrathecal injection of MSC-EVs significantly decreased expression levels of NLRP3, Caspase-1, IL-1β and IL-18 in SDH of IC rats. *n* = 8 per group. *P < 0.05

**Fig. 8:**
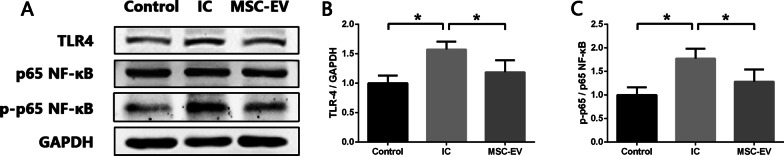
MSC-EVs inhibit activity of TLR4/NF-κB signal pathway in SDH of IC rats. **A**–**C** Western blot analysis showing that expression level of TLR4 and phosphorylation ratio of NF‐κB (p65) were significantly increased in SDH of IC rats compared with normal rats, and intrathecal injection of MSC-EVs significantly decreased expression level of TLR4 and phosphorylation ratio of NF‐κB (p65) in SDH of IC rats. *n* = 8 per group. *P < 0.05

As we have proved that excessive activation of NLRP3 inflammasome played a crucial role in the spinal neuroinflammation of IC, we further verified whether inhibition of NLRP3 inflammasome activation was the underlying mechanism for MSC-EVs to alleviate neuroinflammation and mechanical allodynia in IC rats. As shown in Fig. 7A–E, Western blot analysis revealed that expression levels of NLRP3, Caspase-1, IL-1β and IL-18 were significantly reduced in MSC-EV group compared to IC group. TLR4/NF-κB signal pathway has been proven to regulate NLRP3 inflammasome activation [12], and we investigated the potential effects of MSC-EVs on TLR4/NF‐κB signal pathway in the SDH of IC rats. Western blot results showed that compared with Control group, the expression level of TLR4 and phosphorylation ratio of NF‐κB (p65) were significantly increased in IC group, and MSC-EV treatment caused significant reduction in both of them (Fig. 8A–C). Based on the above data, we concluded that MSC-EVs could inhibit the activity of NLRP3 inflammasome in the SDH of IC rats and TLR4/NF‐κB signal pathway might be the potential regulatory target.

## Discussion

In the present study, we found that neuron-derived NLRP3 inflammasome was activated in the SDH of IC rats, and inhibition of NLRP3 inflammasome using MCC950 alleviated the spinal neuroinflammation and suprapubic mechanical allodynia. In addition, we assessed for the first time the therapeutic efficacy of MSC-EVs in IC rats. After intrathecal injections of MSC-EVs, the mechanical allodynia and frequent micturition of IC rats were significantly alleviated, and the neuroinflammation in SDH was attenuated. Simultaneously, MSC-EVs treatment inhibited the activity of NLRP3 inflammasome as well as TLR4/NF-κB signal pathway. These findings verified our hypothesis that MSC-EVs alleviated neuroinflammation and mechanical allodynia in IC by inhibiting the activation of NLRP3 inflammasome.

The poor efficacy of traditional therapies for IC is predominately due to its complex etiology and pathogenesis [2]. IC used to be considered a bladder-focused disease, while growing evidence reveals that it involves abnormalities in multiple organs or system [5, 32, 39, 40]. Bladder-related pain is the most significant symptom of IC, which shares many characteristics of neuropathic pain [41, 42]. According to inflammation-mediated central sensitization is an important cause of neuropathic pain, our previous work demonstrated that spinal neuroinflammation also participated in the initiation and maintenance of mechanical allodynia in IC [8–10, 32]. When nociceptive information from the bladder is transmitted to SDH, the primary pain center, the afferent nerve endings release excitatory neurotransmitters, such as ATP, substance P, excitatory amino acid, etc., which can activate local glial cells. The proinflammatory cytokines (e.g., TNF-α, IL-1β and IL-6) released by activated glial cells facilitate neuronal excitability and synaptic plasticity, consequently leading to spinal central sensitization and bladder-related hyperalgesia or allodynia [39, 41, 42]. We have previously verified that intrathecal injection of l-α-aminoadipate (an astrocytic specific inhibitor) significantly alleviated the mechanical allodynia in cyclophosphamide-induced IC rats, and as well, IL-1 receptor antagonist exerted a similar effect [10]. These positive results indicated that targeting spinal neuroinflammation is a promising approach for IC therapy.

In order to better understand the regulation mechanism of spinal neuroinflammation in IC and further seek for new therapeutic targets, we explored the role of NLRP3 inflammasome during this process. NLRP3 inflammasome can be activated by a wide range of stimuli and then cleave inactive pro‐IL‐1β and pro‐IL‐18 into mature, active IL‐1β and IL‐18, which initiate multiple signal pathways and drive inflammatory reaction [12]. We detected increased expression levels of NLRP3 inflammasome components (NLRP3 and Caspase-1) as well as IL‐1β and IL‐18 in the SDH of IC rats. After treatment with the NLRP3 inflammasome inhibitor MCC950, expression levels of NLRP3, Caspase-1, IL-1β and IL-18 were significantly reduced. And besides IL-1β and IL-18, levels of TNF-α and IL-6 were also downregulated in the SDH of IC rats. The main sources of these proinflammatory cytokines in the central nervous system are activated microglia and astrocytes [38]. It has been widely discussed that microglia and astrocytes participate actively in the development of inflammatory disorders of central nervous system, such as Alzheimer’s disease [37], multiple sclerosis [43] and spinal cord injury [44]. Our previous studies have detected aberrant activation of microglia and astrocytes in the SDH of IC rats [8]. In the present study, we found that MCC950 significantly decreased the expression levels of IBA-1 (microglia marker) and GFAP (astrocyte marker) in the SDH of IC rats, which meant the activation of microglia and astrocytes was inhibited. These results verified that NLRP3 inflammasome was closely related to the activation of glial cells and development of spinal neuroinflammation in IC.

Given microglia and astrocytes are the core components that mediate neuroinflammation [38], and we have detected that their activity could be inhibited by MCC950, we initially speculated that the activated NLRP3 inflammasomes might be derived from these activated glial cells. However, to our surprise, immunofluorescence co-staining showed that NLRP3 was colocalized scarcely with OX-42 (microglia marker) or GFAP, but predominantly with NeuN (neuron marker), suggesting that neurons were the main source of these NLRP3 inflammasomes in the SDH of IC rats. According to previous reports, NLRP3 was also found to be localized in neurons of SDH in a chronic constriction injury-induced neuropathic pain model [15] and another cancer-induced bone pain model [45], which was consistent with our result. This indicated that neurons also played an important part in the development of spinal neuroinflammation in IC, and we speculated the process might be as follows: when nociceptive information from the bladder was transmitted to SDH, the NLRP3 inflammasomes were assembled and activated in neurons and then triggered the release of IL-1β and IL-18. IL-1β and IL-18 activated adjacent glial cells to release more proinflammatory cytokines, which in return further activated neuron-derived NLRP3 inflammasomes [38]. Thus, the feedback loop among neurons, microglia and astrocytes led to a cascade effect, which subsequently amplified and maintained neuroinflammation in SDH. The effects of MCC950 revealed that by blocking the activation of NLRP3 inflammasome in neurons, this feedback loop could be broken and the neuroinflammation could be restrained.

As our results showed, inhibition of NLRP3 inflammasome using MCC950 effectively alleviated spinal neuroinflammation and mechanical allodynia in IC rats. However, small molecule inhibitors like MCC950 are limited in clinical practice due to their side effects. This motivates us to try to apply MSC-EVs in the treatment of IC. It is well-known that MSCs exert potent anti-inflammatory and immunomodulatory effects through paracrine action [22], and EV is one of the most important paracrine factors [23]. MSC-EVs evoke MSC-like therapeutic effects and their anti-inflammatory activities have been confirmed in various diseases, including inflammatory bowel disease [29], rheumatoid arthritis [30], as well chronic constriction injury-induced neuropathic pain [31]. In the present study, we demonstrated that MSC-EVs also exhibited favorable therapeutic efficacy in IC rats through inflammation modulation. Although a great deal of studies have verified the effectivity of MSCs in the treatment of IC [46], the application of MSC-EVs seems to be more attractive. As a cell-free therapy, MSC-EVs avoid the risk associated with cell transplantation, including immune rejection, endogenous tumor formation and vascular obstruction [23, 26]. More importantly, the processing and storage conditions for EVs are less stringent than MSCs [47], which facilitates standardized mass production and transportation. These advantages bring a bright prospect for the clinical application of MSC-EVs.

Systemic administration by intravenous injection is the most common route of MSC-EVs delivery. Although MSC-EVs can be recruited to the injury site by receptor-mediated interaction, studies have shown that MSC-EVs tend to accumulate in the liver and spleen after systemic injection, resulting in invalid consumption [48, 49]. To achieve sufficient MSC-EVs around the spinal cord and facilitate their direct action on the dysfunctional cells, intrathecal injection was carried out to deliver MSC-EVs in the present study. It is reported that the analgesic effect could be detected as early as 15 min after intrathecal injection of MSC-EVs in a spinal nerve ligation-induced neuropathic pain rat model [31]. In our study, the suprapubic mechanical withdrawal threshold of IC rats was significantly raised after the first intrathecal injection of MSC-EVs, and the additional two doses enhanced the analgesic effect and maintained it until the end of the experiment. In addition, micturition interval of IC rats was also prolonged after MSC-EV treatment. Since frequent micturition of IC is related to the pain caused by bladder filling [1], the prolonged micturition interval further indicated the relief of bladder-related mechanical allodynia. Then as we expected, intrathecal injection of MSC-EVs significantly decreased expression levels of IBA-1 and GFAP, as well as TNF-α and IL-6 in the SDH of IC rats, indicating that MSC-EVs inhibited the activation of microglia and astrocytes in the SDH, contributing to the relief of spinal neuroinflammation in IC rats.

Studies have shown that MSC-EVs regulate the activity of NLRP3 inflammasome in a variety of disease models [50–52]. It has been reported that MSC-EVs inhibited NLRP3 inflammasome activity by delivering circRNA to repair ischemic muscle injury [51]. And in another research, MCS-EVs were proven to ameliorate intervertebral disc degeneration through suppressing NLRP3 inflammasome activation in nucleus pulposus cells [50]. In the present study, we found that MSC-EVs exhibited protective effects similar to MCC950 in IC rats. After intrathecal injection of MSC-EVs, the expression levels of NLRP3, Caspase-1, IL‐1β and IL‐18 in the SDH were reduced, suggesting that MSC-EVs alleviated spinal neuroinflammation of IC by inhibiting the activation of NLRP3 inflammasome. The activation of transcription factor NF-κB is an essential event for NLRP3 inflammasome activation, for it promotes the synthesis of NLRP3, pro‐IL‐1β and pro‐IL‐18 [12]. And TLR4 is an important NF-κB-activating receptor [53]. We assessed the activity of TLR4/NF-κB signal pathway in the SDH of IC rats and found that the expression of TLR4 was upregulated, and phosphorylation ratio of NF‐κB (p65) was also increased which represented a higher transcriptional regulatory activity. While after MSC-EV treatment, expression level of TLR4 and phosphorylation ratio of NF‐κB (p65) showed significant decline. It was indicated that MSC-EVs downregulated the activity of TLR4/NF-κB signal pathway, which might be the regulatory mechanism for MSC-EVs to inhibit activation of neuron-derived NLRP3 inflammasome.

There were inevitably some limitations in our study. MSC-EVs contain large amounts of bioactive proteins and non-coding RNAs [26]. The predominant component and its specific molecular mechanism involved in the inhibition of TLR4/NF-κB signal pathway and NLRP3 inflammasome activation has not been clarified. In addition, further studies are required to elucidate the crosstalk among neurons, microglia and astrocytes after NLRP3 inflammasome activation for a better understanding of the pathogenesis of neuroinflammation in IC.

## Conclusion

The present study proposes a novel strategy for IC treatment based on the application of MSC-EVs. We demonstrate that NLRP3 inflammasome activation is involved in the spinal neuroinflammatory process of IC, and intrathecal injection of MSC-EVs can alleviate neuroinflammation and mechanical allodynia in IC rats by inhibiting NLRP3 inflammasome activation, and TLR4/NF-κB signal pathway may be the potential regulatory target. These findings open up a new avenue for the exploration of IC therapy.

## Data Availability

The datasets supporting the findings of this study are available from the corresponding author on reasonable request.

## Abbreviations

IC: Interstitial cystitis
BPS: Bladder pain syndrome
SDH: Spinal dorsal horn
NLRP3: Nucleotide binding and oligomerization domain-like receptor family pyrin domain-containing 3
MSC: Mesenchymal stem cell
EV: Extracellular vesicle
MSC-EV: Mesenchymal stem cell-derived extracellular vesicle
TNF-α: Tumor necrosis factor alpha
IL-1β: Interleukin 1 beta
IL-6: Interleukin 6
IL-18: Interleukin 18
NeuN: Neuronal nuclei
IBA-1: Ionized calcium binding adapter molecule 1
GFAP: Glial fibrillary acidic protein
TLR4: Toll-like receptor 4
NK-κB: Nuclear factor kappa B
TEM: Transmission electron microscopy
DLS: Dynamic light scattering
ALIX: Apoptosis linked gene 2 interacting protein X
